# Active thermal cloaking and mimicking

**DOI:** 10.1098/rspa.2020.0941

**Published:** 2021-05

**Authors:** Maxence Cassier, Trent DeGiovanni, Sébastien Guenneau, Fernando Guevara Vasquez

**Affiliations:** ^1^ Aix Marseille Univ, CNRS, Centrale Marseille, Institut Fresnel, Marseille, France; ^2^ University of Utah, Mathematics Department, Salt Lake City, UT 84112, USA; ^3^ UMI 2004 Abraham de Moivre-CNRS, Imperial College London, London, SW7 2AZ, UK

**Keywords:** heat equation, active cloaking, potential theory, green identities

## Abstract

We present an active cloaking method for the parabolic heat (and mass or light diffusion) equation that can hide both objects and sources. By active, we mean that it relies on designing monopole and dipole heat source distributions on the boundary of the region to be cloaked. The same technique can be used to make a source or an object look like a different one to an observer outside the cloaked region, from the perspective of thermal measurements. Our results assume a homogeneous isotropic bulk medium and require knowledge of the source to cloak or mimic, but are in most cases independent of the object to cloak.

## 1. Introduction

**Figure 1.**
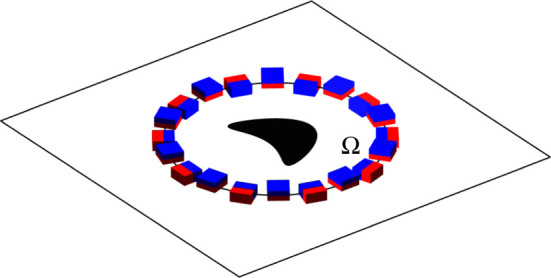
An illustrative example of an arrangement of Peltier elements that could be used to cloak objects (e.g. a kite) inside a two-dimensionalregion Ω, illustrated here by a disk within a heat-conducting plate. Each Peltier device is represented by two adjacent red and blue boxes, where the heat flux it can create is oriented in the direction normal to their interface. We have represented both Peltier elements that transport heat within the plate (across the boundary ∂Ω of Ω) and also between the exterior and the plate. (Online version in colour.)

The concept of invisibility cloaks was originally introduced in optics and electromagnetics [1–4] to illustrate a method to manipulate fields using passive anisotropic heterogeneous media deduced from a geometric transform of the governing equations. This passive cloaking approach was adapted to diffusion equations [5]. Here, we consider a similar problem, but instead of using a passive medium for the cloak, we use specially designed heat sources and sinks distributed on a surface surrounding the object. Using active sources rather than a passive material has the advantage of being tunable, which is especially desirable in the transient regime. Tunability also allows us to solve other problems such as cloaking a source or sink of heat and making a source, sink or object appear as another one (the mimicking problem). Such active sources can be realized physically using heat pumps such as Peltier elements, which use electricity to move heat across a metal-metal junction (e.g. [6] and figure 1). Active sources for this ‘interior cloaking of an object’ problem have already been proposed and demonstrated experimentally for the steady-state heat equation (Poisson equation using polar coordinates) [7,8], where under a steady-state temperature distribution, Peltier elements are used to dissimulate either a circular hole in a conductive plate or a penetrable inclusion with a different constant conductivity. To deal with time-varying solutions to the heat equation we envision a set-up similar to [7,8], but with Peltier devices that are either used to transport heat within the plate or act as heat sources/sinks on the plate by transporting heat between the environment and the plate, as illustrated in figure 1. An alternative route using a single active dipole source placed inside the object to cloak in a constant gradient steady-state regime is proposed in [9].

### (a) Problem set-up

**Figure 2.**
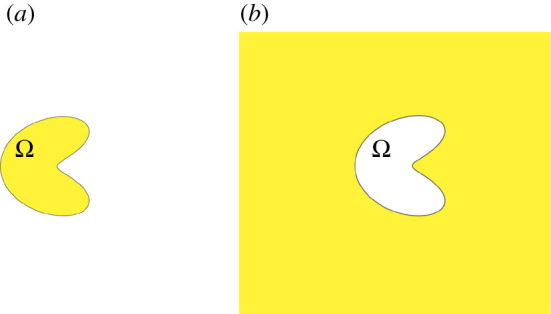
In (*a*), we illustrate the ‘interior reproduction problem’, which consists of reproducing a solution to the heat equation in the interiorof a bounded region Ω (in yellow), while enforcing a zero solution outside of $\bar{\Omega }$ (in white) by placing heat sources on the boundary ∂Ω. In (*b*), we illustrate the ‘exterior reproduction problem’ in a similar way. (Online version in colour.)

Mathematically, we view the cloaking problem as reproducing certain solutions to the heat equation inside or outside a closed surface by a dipole surface distribution determined by the temperature at the surface (Dirichlet trace) and a monopole surface distribution determined by its flux (Neumann trace), via the Green identities. In particular, given a solution to the heat (or mass or light diffusion) equation in a homogeneous medium and with no sources inside of a domain, it is possible to reproduce it inside the domain with a distribution of sources on the surface of the domain, while also giving a zero solution outside. We call this the *interior reproduction problem*, see figure 2*a*. Similarly, the *exterior reproduction problem* is to reproduce a solution to the heat equation in an *unbounded* homogeneous medium with no sources outside of a domain, while keeping a zero solution inside the domain (figure 2*b*). As we shall see, a growth condition for the heat equation solution is needed to guarantee that the exterior reproduction problem can be solved. This growth condition plays a role similar to a radiation boundary condition for the Helmholtz equation (§2.).

By combining solutions to interior/exterior reproduction problems, we can achieve cloaking or mimicking for the heat equation in the following scenarios.

**Interior cloaking of a source**: Given a localized heat source, find an active surface or cloak surrounding the source so that the source cannot be detected by thermal measurements outside the cloak (§3.a).

**Interior cloaking of an object**: Given a passive object (e.g. an inclusion), find an active surface or cloak surrounding the object so that the temperature distribution outside the cloak is indistinguishable from having a region of homogeneous medium instead of the cloak and the object (§3.b).

**Source mimicking problem:** Given a localized source, find an active surface or cloak surrounding it so that the source appears as a different source for an observer outside the cloak (§4.a).

**Object mimicking problem:** Given a passive object inclusion, find an active surface or cloak surrounding it so that the object appears as a different object for an observer outside the cloak (§4.b).

Because we are using source distributions, we can achieve cloaking of inclusions and sources, regardless of how complicated they are and on arbitrarily large time intervals. One drawback of our method is that the source distribution completely surrounds the object or source that we want to cloak or mimic. Another drawback is that we assume perfect knowledge of the fields to reproduce on a surface, though we demonstrate numerically that there is some robustness to noise (§2.c).

### (b) Related work in active cloaking and mimicking

The idea of using active sources based on the Green identities to cloak objects was first proposed for waves by Miller [10]. The way of finding the sources for active cloaking is similar to that in active sound control for e.g. noise suppression [11,12]. One problem with this approach is that the sources completely surround the cloak. However, only a few sources are needed to cloak as was shown in [13–18] for the Laplace and Helmholtz equations. The approach can be extended to elastic waves [19] and flexural (plate) waves [20,21]. The active cloaking approach can be applied to the steady-state diffusion equation with the role of sources and sinks over a thick coating played by chemical reactions [22]. Active cloaking has been demonstrated experimentally for the Laplace equation [23,24], electromagnetics [25] and the steady-state heat equation [7,9]. Finally, the illusion/mimicking problem was first proposed using metamaterials via a transformation optics approach [26] and then with active exterior sources [27].

### (c) Applications

Although all the results are presented in the context of the heat equation, they also apply to the diffusion equation, which can be used to model e.g. diffusion of a species in a porous medium. In this case, the active surface would consist of pumps that can transport the species either across the medium or between the medium and the environment. For the diffusion equation case, we can either hide or imitate a source or an inclusion with different diffusivity properties.

Controlling the heat flux may find applications in enhancing the efficiency of thermal devices in solar thermal collectors, protecting electronic circuits from heating, or the design of thermal analogues of electronic transistors, rectifiers and diodes [28]. Moreover, all our results could be easily adapted to control of mass diffusion with potential applications ranging from biology with the delay of the drug release for therapeutic applications [29,30] to civil engineering with the control of corrosion of steel in reinforced concrete structures [31]. We further note that in many media, such as clouds, fog, milk, frosted glass or media containing many randomly distributed scatterers, light is not described by the macroscopic Maxwell equations, but rather by Fick’s diffusion equation as photons of visible light perform a random walk. Cloaking for diffusive light was experimentally achieved in [32] using the transformed Fick’s equation [29] and this suggests potential applications of our work in control of diffusive light as well.

### (d) Other thermal cloaking approaches

Apart from the active cloaking strategies for the steady-state heat equation in [7,9], there are passive cloaking methods for the heat equation that use carefully crafted materials to hide objects [5,29,33–35]. Such materials, whose effective conductivity mimics that in the heat equation after a suitable change of variables has been made, are quite bulky. In [35], some proof of concept of passive thermal cloaking was achieved with a metamaterial cloak consisting of 10 concentric layers mixing copper and polydimethylsiloxane in a copper plate. However, it has been numerically shown using homogenization in [36] that one would require over 10 000 concentric layers with an isotropic homogeneous conductivity to accurately mimic the required anisotropic heterogeneous conductivity within a thermal cloak in order to achieve some markedly improved cloaking performance in comparison with [35].

### (e) Outline

We start in §2. by recalling results on representing solutions to the heat equation by surface integrals. This section includes a growth condition on heat equation solutions that is sufficient to ensure that the exterior reproduction problem is solvable and numerical experiments illustrating both the interior and exterior reproduction problems in two dimensions. We also underline properties related to the maximum principle of the heat equation that we use on one hand to point out some stability of the two reproduction problems and on the other hand to interpret the numerical error in our simulations. Then in §3. we explain how to cloak a source (§3.a) or an object (§3.b). The mimicking problem is presented in §4. for both mimicking objects (§4.b) and sources (§4.a). The numerical method we use to illustrate our approach in two dimensions is explained in §5. Finally, our results are summarized in §6.

## 2. Integral representation of heat equation solutions

We start by recalling results on boundary integral representation of solutions to the heat equation. Concretely we show in §§2.a and 2.b how to use a distribution of monopole and dipole heat sources on a closed surface, an ‘active surface’, to reproduce a large class of solutions to the heat equation inside and outside the surface. We include a two-dimensional numerical study (§2.c) of how the reproduction error is affected by the time step, the boundary discretization and errors in the boundary density we use to represent the fields.

The temperature *u*(*x*, *t*) of a homogeneous isotropic body satisfies the heat equation

2.1 $$\rho c\frac{\partial u}{\partial t}=\kappa \Delta u+\tilde{h},\;\text{for}\;t>0,$$

where *u* is in Kelvin, $x\in R^{d}$ is the position in metres (*d* ≥ 1 is the dimension), *t* is the time in seconds, *κ* is the thermal conductivity (W m^−1^ K^−1^), *c* is the specific heat (JK^−1^ kg^−1^), *ρ* is the mass density (kg m^−3^), $\tilde{h}(x,t)$ is a source term (W m^−3^) and $\Delta =\sum_{i=1}^{d}\partial^{2}/\partial x_{i}^{2}$ is the Laplacian operator. To simplify the exposition, we consider instead

2.2 $$\frac{\partial u}{\partial t}=k\Delta u+h,\;\text{for}\;t>0,$$

where *k* = *κ*/*ρc* is the thermal diffusivity (m^2^ s^−1^) and $h=\tilde{h}/\rho c$ is the source term (K s^−1^). In dimension *d*, the Green function or heat kernel for (2.2) is

2.3 $$K(x,t)=\left\{ \begin{array}{ll} {\left(4\pi kt\right)}^{-d/2}\exp[-|x{|}^{2}/4kt], & \text{for}\;t>0\;\text{and}\;x\in R^{d},\\ 0, & \text{otherwise}, \end{array}\right.$$

where | · | is the Euclidean norm in $R^{d}$. We point out that outside the origin (*x*, *t*) = (0, 0), *K*(*x*, *t*) is a smooth function even on the line *t* = 0. This can be shown directly or by using the hypoellipticity property of the heat operator. Namely, as the heat kernel solves the homogeneous heat equation in the distributional sense on any open set that does not contain the origin, by hypoellipticity (see [37] theorem 1.1 p. 192) *K*(*x*, *t*) is a *C*^∞^ function on $\left(R^{d}\times R)-\{(0,0)\right\}$. Thus *K*(*x*, *t*) is a smooth solution of the heat equation on any open subset of this set.

We consider a bounded non-empty open set Ω in $R^{d}$, *d* ≥ 1 with a finite number of connected components (with disjoint closures) and for *d* ≥ 2, we assume that its boundary ∂Ω is Lipschitz continuous. The interior reproduction problem (§2.a) is to reproduce a solution of the homogeneous heat equation in the space–time cylinder Ω×(0,∞) by placing appropriate source densities on its boundary ∂Ω×[0,∞) and possibly on Ω×{0} (initial condition). The sources are chosen such that the fields vanish in $\left(R^{d}-\bar{\Omega })\times (0,\infty \right)$ (where $\bar{\Omega }=\Omega \cup \partial \Omega$ denotes the closure of Ω). If the initial condition is harmonic, it is sufficient to have sources on ∂Ω×[0,∞) only. For the exterior reproduction problem (§2.b), we seek to reproduce a solution of the homogeneous heat equation in $\left(R^{d}-\bar{\Omega })\times (0,\infty \right)$ using sources on ∂Ω×[0,∞) and possibly on $\left(R^{d}-\bar{\Omega })\times \{0\right\}$. The fields are required to vanish in Ω×(0,∞).

### (a) Reproducing fields in the interior of a bounded region

The goal here is to reproduce solutions *u* to (2.2) in Ω by controlling sources on ∂Ω while leaving the exterior unperturbed. More precisely, for some temperature field *u*(*x*, *t*), we wish to generate $u_{\Omega }(x,t)$ such that for *t* > 0:

2.4 $$u_{\Omega }(x,t)=\left\{ \begin{array}{ll} u(x,t), & x\in \Omega ,\\ 0, & x\notin \bar{\Omega }. \end{array}\right.$$

Note that $u_{\Omega }(x,t)$ is not defined for x∈∂Ω, as usual in boundary integral equations. For initial condition *u*(*x*, 0) = *f*(*x*), x∈Ω and a source term *h*(*x*, *t*) spatially supported in $R^{d}-\Omega$, this can be achieved via the Green identities (see e.g. [38–40])

2.5 $$\begin{array}{rl} u_{\Omega }(x,t) & =\int_{0}^{t}\;\text{d}s\int_{\partial \Omega }\;\text{d}S(y)\left[\frac{\partial u}{\partial n}(y,s)K(x-y,t-s)-u(y,s)\frac{\partial K}{\partial n}(x-y,t-s)\right]\\ & \;+\int_{\Omega }\;\text{d}y\;f(y)K(x-y,t),\;t>0, \end{array}$$

here *n*(*y*) is the outward pointing unit length normal vector at y∈∂Ω. The Green identities guarantee that $u_{\Omega }(x,t)=0$ for $x\notin \bar{\Omega }$, as desired. The first term in the integral (2.5) is the single layer potential (a collection of monopole heat sources) and the second is the double layer potential (a collection of dipole heat sources). For zero initial conditions, the solution *u* is completely represented within Ω by the single and double layer potentials, with densities given by the field to be reproduced and its normal derivative on ∂Ω. For *d* = 1, the measure *S* is a sum of Dirac measures supported at the finite collection of points in ∂Ω, i.e. $S=\sum_{x\in \partial \Omega }\delta_{x}$.

We point out that the representation formula (2.5) holds for instance if $u\in C^{\left(2,1\right)}(\bar{\Omega }\times [0,+\infty ))$. For a non-empty open subset O of $R^{d}$ (with Lipschitz continuous boundary ∂O), the space $C^{\left(2,1\right)}(\bar{O}\times [0,+\infty ))$ denotes here the space of $C^{1}(\bar{O}\times [0,+\infty ))$ scalar real-valued functions of the variables (*x*, *t*) whose second order spatial partial derivatives in *x* exist on O×(0,+∞) and can be continuously extended to $\bar{O}\times [0,+\infty )$. We recall that in this setting for an integer *k* ≥ 0, $C^{k}(\bar{O}\times [0,+\infty ))$ is the space of scalar real-valued functions of (*x*, *t*) that are *k*-times continuously differentiable on the open set O×(0,+∞) and whose partial derivatives of order *α* with 0 ≤ *α* ≤ *k* can be continuously extended to $\bar{O}\times [0,+\infty )$. Thus the space $C^{\left(2,1\right)}(\bar{O}\times [0,+\infty ))$ contains in particular $C^{2}(\bar{O}\times [0,+\infty ))$ but is less restrictive since no conditions are imposed on second order time and space–time partial derivatives by the heat equation.

**Remark 2.1. (Sobolev regularity)**

A less restrictive condition on the regularity of the solution *u* in the integral representation (2.5) is to assume some Sobolev regularity. More precisely, one introduces for a bounded open set $O\subset R^{d}$ and a time *T* > 0, the anisotropic Sobolev spaces

2.6 $$H^{r,s}(O\times (0,T)):=L^{2}((0,T),H^{r}(O))\cap H^{s}((0,T),L^{2}(O))\;\text{ for }r,s\geq 0$$

endowed with their standard Sobolev norm, see [37,39,41] for more details. In this setting, for Lipschitz domain O, one defines ‘the Boundary Sobolev space’ $H^{1/2,1/4}(\partial O\times (0,T))$ as in (2.6) by replacing O by ∂O. In ([39], lemma 2.4), the author proves that $H^{1/2,1/4}(\partial O\times (0,T))$ is exactly the trace space of $H^{1,1/2}(O\times (0,T))$ functions on ∂O×(0,T) with zero initial condition, i.e. whose trace vanishes at the initial time *t* = 0, i. e. on O×{0}. They introduce also $H^{-1/2,-1/4}(\partial O\times (0,T))$ as the dual space of $H^{1/2,1/4}(\partial O\times (0,T))$ and denote by 〈 · , · 〉 the duality pairing of these two spaces.

If one assumes that $u\in H^{1,1/2}(\Omega \times (0,T))$ for some *T* > 0 and *Lipschitz* boundary ∂Ω is a solution of the heat equation (2.2) (in the distributional sense) with zero initial condition (*f* = 0) and a source term spatially supported in $R^{d}-\Omega$, then (see [39], theorem 2.20) the formula (2.5) for $u_{\Omega }$ defined by (2.4) and *t* ∈ (0, *T*) can be rewritten as:

2.7 $$u_{\Omega }(x,t)=\left\langle \frac{\partial u}{\partial n},K(x-\cdot ,t-\cdot )\right\rangle -\left\langle \frac{\partial K}{\partial n}(x-\cdot ,t-\cdot ),u\right\rangle ,$$

where $\partial u/\partial n\in H^{-1/2,-1/4}(\partial \Omega \times (0,T))$, $u\in H^{1/2,1/4}(\partial \Omega \times (0,T))$. Moreover, as (y,s)↦K(x−y,t−s) is *C*^∞^ −smooth functions on a vicinity of ∂Ω×[0,T] (since x∉∂Ω), it is clear that $K(x-\cdot ,t-\cdot )\in H^{1/2,1/4}(\partial \Omega \times (0,T))$ and $\partial K/\partial n(x-\cdot ,t-\cdot )\in L^{2}(\partial \Omega \times (0,T))$. Thus the second duality pairing in (2.7) is a regular integral because $\partial K/\partial n(x-\cdot ,t-\cdot )\in L^{2}(\partial \Omega \times (0,T))$. If one considers now that ∂Ω is *C*^2^ (so more smooth than Lipschitz continuous) and *u* is slightly more regular, namely in $H^{2\alpha ,\alpha }(\partial \Omega \times (0,T))$ for *α* > 3/4, then one has $\partial u/\partial n\in L^{2}(\partial \Omega \times (0,T))$. Thus, in this latter configuration, the duality pairings in (2.7) are replaced by inner products on $L^{2}(\partial \Omega \times (0,T))$ as in (2.5) (with *f* = 0). This is explained in more detail in remark 2.7.

**Remark 2.2. (Causality and instantaneous control)**

The boundary integral representation (2.5) is *causal* in the sense that to reproduce $u_{\Omega }(x,t)$, *t* > 0, we only need information about *u* in the past, i.e. for times before the present time *t*. Moreover, the time convolution (2.5) suggests that at the present time *t*, we only require control of heat sources localized in time to the present time *t*. Indeed, the integral over ∂Ω in (2.5) is a collection of monopole and dipole sources localized in time to *s* and that depends only on knowing *u* and ∂*u*/∂*n* at time *s*. Moreover, the contribution of past *s*, i.e. with *s* < *t*, amounts to the memory effect of the bulk. Thus for experimental purposes, the boundary integral representation could be approximated by e.g. Peltier devices.

**Figure 3.**
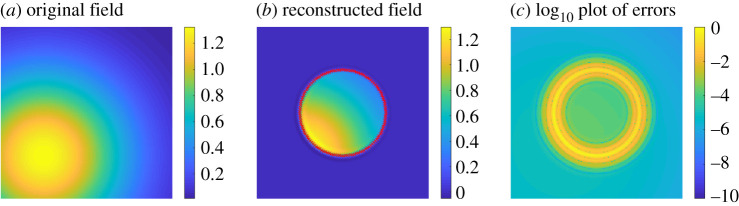
Numerical example of the interior field reproduction problem for a point source located at (1/4, 1/4) and *t* = 0 s. A snapshot of the original field at time *t* = 0.2 s appears in (*a*). The field is reconstructed inside a disk of radius 1/4 centred at (1/2, 1/2) by using only heat sources on the corresponding circle (*b*). In (*c*), we show the log _10_ of the reconstruction error (the absolute value of the difference between the exact $u_{\Omega }$ and its numerical approximation). We generated a plot similar to (*c*) by taking the maximum over the time interval [0.2, 0.3] at each grid point (the plot being very similar to (*c*), we include the code to generate it in [42]). This indicates that the maximum error is attained near ∂Ω in space (and in time at *t* = 0.2 on the time interval [0.2, 0.3]), conforming to the maximum principle (applied with initial time *t* = 0.2) for the interior problem (remark 2.3) and the exterior one (remark 2.9). We can use the maximum principle on the computed fields because the numerical method we use generates solutions to the heat equation (see §5. for more details). (Online version in colour.)

A numerical example is given in figure 3. Here, the field *u* is generated by a point source at *x* = (0.25, 0.25) and *t* = 0. For the heat equation, we took *k* = 0.3 and the domain is $\Omega =B(x_{0},r_{0})$, the open ball with centre *x*_0_ = (0.5, 0.5) and radius *r*_0_ = 0.25. It should be noted that in all of the numerical examples the thermal diffusivity *k* is chosen for the convenience of computations, and may be different depending on the numerical experiment. We computed the fields on the unit square [0, 1]^2^ with a 200 × 200 uniform grid at time *t* = 0.2 s. The integral (2.5) is approximated using the midpoint rule in time with 200 equal length subintervals of [0, *t*] and the trapezoidal rule on ∂Ω with 128 uniformly spaced points. A more detailed explanation of the numerical method appears in §5. The accuracy of our numerical method with respect to discretization changes and noise is evaluated in §2.c. Figure 3*c* shows a plot of the log _10_ error between the computed field and the desired one. It can be observed that the accuracy of the numerical method improves as we move away from ∂Ω.

We point out that the term in (2.5) involving the initial condition is very different from the other terms because it is an integral over Ω rather than just the boundary ∂Ω. If the initial condition *f*(*x*) is non-zero but harmonic, this integral over Ω can also be expressed as an integral over ∂Ω by using Green identities as follows (see [38])

2.8 $$\int_{\Omega }\;\text{d}y\;f(y)K(x-y,t)=\int_{\partial \Omega }\;\text{d}S(y)\left[f(y)\frac{\partial \phi }{\partial n}(x-y,t)-\phi (x-y,t)\frac{\partial f}{\partial n}(y)\right],$$

where *ϕ*(*x*, *t*) is given in two dimension by [38]:

$$\phi (x,t)=\frac{-1}{4\pi }\mathrm{Ein}\left(\frac{|x{|}^{2}}{4kt}\right),$$

where Ein⁡(z)=E⁡(z)+ln⁡z+γ, *γ* is the Euler constant and

$$E(z)=\int_{z}^{+\infty }\frac{{\text{e}}^{-\zeta }}{\zeta }\;\text{d}\zeta$$

is the exponential integral (see [43], eqns 6.2.2 and 6.2.4). The expression for *ϕ* in three dimensions is given in [38]. From the experimental perspective, it is not clear whether the kernels in the boundary integrals (2.8) can be achieved with heat monopole and dipole sources. So we assume from now on that the initial condition is harmonic but does not need to be reproduced using sources on ∂Ω. Under this assumption, we can simply subtract the initial condition to obtain the heat equation with zero initial condition, which is the case that we focus on. Numerical experiments illustrating a non-zero initial condition are included in electronic supplementary material, appendix B.

**Remark 2.3.**

As formula (2.5) uses the boundary data and the initial condition to express $u_{\Omega }$, this reconstruction of *u* satisfies some stability due to the maximum principle applied to the heat equation (this property is shared by a large class of elliptic and parabolic equations, see [44], but does not hold in general for hyperbolic equations such as the wave equation). Indeed, for any *T* > 0, the maximum principle [44–47] states that a continuous function on $\bar{\Omega }\times [0,T]$ that solves the homogeneous heat equation (2.2) on Ω×(0,T) (in the distributional sense) reaches its minimum and maximum either at *t* = 0 or at any time *t* ∈ [0, *T*] on the boundary ∂Ω. For instance, the solution of the initial-Dirichlet boundary value problem (with no sources) described in ch. 7 pp. 171–172 of [47] satisfies the above conditions. In particular, this solution is continuous up to the boundary, i.e. on $\bar{\Omega }\times [0,T]$. To obtain such continuity, the continuous initial data and the continuous Dirichlet data have to match on ∂Ω at *t* = 0, e.g. [47].

To understand the stability, we take two solutions $u_{j}\in C^{0}(\bar{\Omega }\times [0,T])$, *j* = 1, 2, which satisfy the homogeneous heat equation (2.2) in Ω×(0,T) for *T* > 0 in the distributional sense. In this setting, solutions in the distributional sense are also smooth solutions of the homogeneous heat equation (2.2) (since by hypoellipticity, $u_{j}\in C^{\infty }(\Omega \times (0,T))$ for *j* = 1, 2, e.g. [37] theorem 1.1 p. 192). Thus, one can apply the maximum principle (see [45], theorem 10.6 p. 334) to obtain that

2.9 $$\max_{\bar{\Omega }\times [0,T]}|u_{2}(x,t)-u_{1}(x,t)|=\max_{\left(\Omega \times \{0\})\cup (\partial \Omega \times [0,T]\right)}|u_{2}(x,t)-u_{1}(x,t)|.$$

Moreover, if the initial conditions are harmonic, using the maximum principle for the Laplace equation gives

2.10 $$\max_{\bar{\Omega }\times [0,T]}|u_{2}(x,t)-u_{1}(x,t)|=\max_{\partial \Omega \times [0,T]}|u_{2}(x,t)-u_{1}(x,t)|.$$

Thus, in the space of solutions of the homogeneous heat equation (with the regularity described above), an error committed on the initial condition *u*(*x*, 0) or the boundary Dirichlet data of a solution *u* (i.e. the dipole distribution) on ∂Ω×[0,T] controls the error (in the supremum norm) in the reconstruction of $u_{\Omega }$ in Ω×(0,T].

Finally, we point out an important property that constrains the behaviour of *u*_2_ − *u*_1_ if the maximum in (2.9) is attained at a point not located at the boundary or at the initial time. Under the additional assumption that Ω is connected, one shows based on the mean value property of the heat operator and a connexity argument (as in [46], §2.2.3, theorem 4 pp. 54–55), that if there exists a point $(x_{0},t_{0})\in (0,T]\times \Omega$ such that |*u*_2_ − *u*_1_| reaches its maximum at (*x*_0_, *t*_0_) in (2.9), then *u*_2_ − *u*_1_ has to be constant in $\bar{\Omega }\times [0,t_{0}]$. Indeed, for formula (2.10), this property holds also for *t*_0_ = 0 since the initial condition is harmonic and the Laplace operator satisfies a mean value property.

### (b) Reproducing fields exterior to a bounded region

For the exterior reproduction problem, we seek to reproduce solutions to (2.2) outside of $\bar{\Omega }$ by controlling sources on ∂Ω, while leaving the interior unperturbed. That is, for some temperature field *v*(*x*, *t*) solving the heat equation we wish to generate $v_{\Omega }(x,t)$, such that

2.11 $$v_{\Omega }(x,t)=\left\{ \begin{array}{ll} 0, & x\in \Omega ,\\ v(x,t), & x\notin \bar{\Omega }, \end{array}\right.$$

for *t* > 0. Note that we have used the subscript Ω differently in (2.11) than in (2.4). We adhere to the convention that $u_{\Omega }$ always refers to the interior reproduction problem of heat equation solution *u* and $v_{\Omega }$ refers to the exterior reproduction of a field *v*. The problem of reproducing $v_{\Omega }$ can be solved when the source term associated with *v*(*x*, *t*) is spatially supported in $\bar{\Omega }$ for *t* ≥ 0. We only consider the case where *v*(*x*, 0) = 0. Non-zero initial conditions are left for future studies.

Without further assumptions the exterior reproduction problem may not have a unique solution as can be illustrated by the one-dimensional non-uniqueness example by Tychonoff [48]. Uniqueness for the Dirichlet problem can be guaranteed via the maximum principle ([46], ch. 2, §3, theorem 7) (see also remark 2.9) or via Sobolev regularity estimates in space and time [41,49]. For the transmission problem, growth restrictions in the Laplace domain are used to prove uniqueness in [50]. Here, we want to establish a boundary representation formula for exterior solutions to the heat equation, which uses both Dirichlet and Neumann data. Such exterior representation formula has already been mentioned in [41,50,51], but without giving an explicit growth condition on the heat equation solution that guarantees its validity. We give a growth condition for the heat equation, analogous to the Sommerfeld radiation condition for the Helhmholtz equation [52] (a comparison between these two conditions is in remark 2.5).

**Figure 4.**
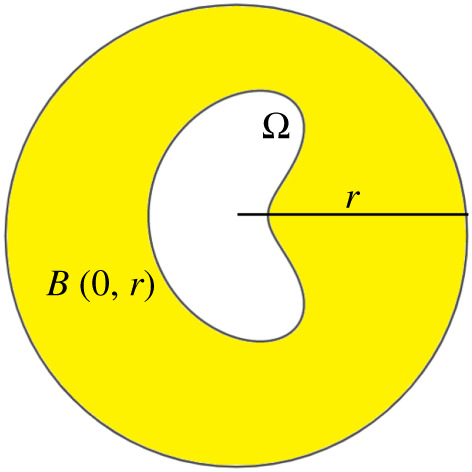
To show that the exterior reproduction problem has a solution outside of a bounded region Ω, we use the interior reproductionproblem in the yellow region. Theorem 2.6 shows that for fields satisfying condition 2.4, the contribution of the sources on the sphere *S*^*d*^(0, *r*) of radius *r* vanishes as *r* → ∞ for *d* ≥ 1. (Online version in colour.)

To prove a boundary potential formula for the exterior reproduction problem, we follow the same steps as in [52] for the Helmholtz equation. Namely, we use the interior reproduction problem (§2.a) on the complement of $\bar{\Omega }$ truncated to a ball of sufficiently large radius *r* (figure 4), and then give a sufficient condition guaranteeing that the contribution from the sources at |*x*| = *r* vanishes as *r* → ∞, allowing heat equation solutions outside of $\bar{\Omega }$ to be reproduced by only controlling sources at ∂Ω. The sufficient condition that we impose on the growth of *v*(*x*, *t*) is close to the growth condition for the exterior Dirichlet problem uniqueness, see remark 2.5.

**Condition 2.4. (Growth condition)**

Let *d* ≥ 1. A function *v*(*x*, *t*) is said to satisfy the ‘growth condition’ if there exists an *r*_0_ > 0 such that *v* is differentiable on $\left(R^{d}-\bar{B(0,r_{0})})\times (0,+\infty \right)$ and satisfies for *r* > *r*_0_:

2.12 $$\left|\frac{\partial v}{\partial n}(r\xi ,t)+\left(\frac{2r}{4kt}\right)v(r\xi ,t)\right|\leq Cr^{m}\;{\text{e}}^{a\;r^{b}},\;\forall t>0,\;\xi \in S^{d}(0,1),$$

where *m* is an integer, *C* > 0, *a* ≥ 0 are constants, the exponent *b* satisfies 0 ≤ *b* < 2, *S*^*d*^(0, 1) is the sphere of radius 1 centred at the origin in *d* dimensions and *n* = *ξ* is the outward normal vector at *x* = *rξ*.

**Remark 2.5.**

The bound in condition 2.4 is not as restrictive as the Sommerfeld radiation condition for the Helmholtz equation because of the Gaussian spatial decay of the heat kernel at fixed positive time. Indeed, the Green function for the Helmholtz equation decays in space faster than *r*^−*m*^ as *r* → ∞, where *m* > 0 depends on the dimension and *r* = |*x*|. On the other hand, the heat kernel decays faster than $r^{m}\;{\text{e}}^{-a\;r^{b}}$ as *r* → ∞, with *a* > 0, 0 ≤ *b* < 2 and m∈Z. This is how we motivate the bound in condition 2.4. However, we conjecture that it may be possible to improve the bound to allow *b* = 2 (Gaussian decay over a polynomial), which would bring it on the same par as the growth condition guaranteeing uniqueness for the Dirichlet problem outside of a bounded domain (remark 2.9). We point out that condition 2.4 is satisfied by a large class of solutions of the heat equation (2.1) in $\left(R^{d}-\bar{\Omega })\times (0,+\infty \right)$ that includes in particular the heat kernel and any of its spatial derivatives (see lemma 2.8).

**Theorem 2.6.**

*Let* $v\in C^{\left(2,1\right)}((R^{d}-\Omega )\times [0,+\infty ))$ *be a solution to the heat equation* (2.1) *in* $\left(R^{d}-\bar{\Omega })\times (0,+\infty \right)$ *for* *d* ≥ 1, *with zero initial condition and a source term spatially supported in the compact set* $\bar{\Omega }$. *Furthermore, assume that* *v* *satisfies the growth condition 2.4, then* *v* *can be reproduced for* *t* > 0 *in the exterior of* $\bar{\Omega }$ *by the boundary representation formula*

2.13 $$v_{\Omega }(x,t)=-\int_{0}^{t}\;\text{d}s\int_{\partial \Omega }\;\text{d}S(y)\left[\frac{\partial v}{\partial n}(y,s)K(x-y,t-s)-v(y,s)\frac{\partial K}{\partial n}(x-y,t-s)\right],$$

*where* $v_{\Omega }$ *is as in* (2.11).

**Proof.**

Let $\left(x,t)\in (R^{d}-\bar{\Omega })\times (0,+\infty \right)$ be fixed. Without loss of generality, we assume that 0∈Ω and take *r* to be large enough such that $\bar{\Omega }\subset B(0,r)$ and $x\in B(0,r)-\bar{\Omega }$. Since $v\in C^{\left(2,1\right)}((R^{d}-\Omega )\times [0,+\infty ))$ satisfies the heat equation with a zero source term outside of $\bar{\Omega }$, we can use (2.5) with zero initial condition to reproduce *v*(*x*, *t*) on the bounded open set $B(0,r)-\bar{\Omega }$, giving

2.14 $$v_{B(0,r)-\bar{\Omega }}(x,t)=I_{1}(x,t;r)+I_{2}(x,t),$$

where

2.15 $$\begin{array}{rl} I_{1}(x,t;r) & =\int_{0}^{t}\;\text{d}s\int_{S(0,r)}\;\text{d}S(y)\left[\frac{\partial v}{\partial n}(y,s)K(x-y,t-s)-v(y,s)\frac{\partial K}{\partial n}(x-y,t-s)\right]\\ \text{and}\;\;I_{2}(x,t) & =-\int_{0}^{t}\;\text{d}s\int_{\partial \Omega }\;\text{d}S(y)\left[\frac{\partial v}{\partial n}(y,s)K(x-y,t-s)-v(y,s)\frac{\partial K}{\partial n}(x-y,t-s)\right]. \end{array}$$

The minus sign in *I*_2_(*x*, *t*) is because we defined *n* as the outward pointing unit normal to Ω. The goal is now to show that *I*_1_(*x*, *t*;*r*) → 0 as *r* → ∞, leaving us with only *I*_2_(*x*, *t*), which gives the desired result (2.13). We rewrite *I*_1_(*x*, *t*;*r*) using

$$\frac{\partial K}{\partial n}(x,t)=K(x,t)\left(\frac{-2x}{4kt}\cdot n\right),$$

and switching the convolutions in time to get

2.16 $$I_{1}(x,t;r)=\int_{0}^{t}\;\text{d}s\int_{S(0,r)}\;\text{d}S(y)\left[\frac{\partial v}{\partial n}(y,t-s)+\left(\frac{2y}{4k(t-s)}\cdot n\right)v(y,t-s)\right]K(x-y,s).$$

We define *ξ* = *x*/|*x*| (*x* ≠ 0 since $x\notin \bar{\Omega }$). Thus, for *y* ∈ *S*(0, *r*), we can bound the heat kernel by

2.17 K(x−y,s)≤K(x−rξ,s),

because |*x* − *y*| ≥ |*x* − *rξ*| holds for |*y*| = *r*. Noticing that we also have *y* · *n* = *r* for |*y*| = *r*, we can use condition 2.4 to bound *I*_1_(*x*, *t*;*r*) for sufficiently large *r*. Thus, using (2.16), the bound (2.17) and applying condition 2.4 leads to

2.18 $$|I_{1}(x,t;r)|\leq Cr^{m}\;{\text{e}}^{a\;r^{b}}A_{d}(r)\;\int_{0}^{t}\;\text{d}s\;\frac{1}{{\left(4\pi ks\right)}^{d/2}}\;{\text{e}}^{-|x-r\xi {|}^{2}/4ks},$$

where *A*_*d*_(*r*) is the surface of a sphere of radius *r* in *d* dimensions, which is given in terms of the Gamma function (e.g. [43], eqn 5.2.1) by

$$A_{d}(r)=\frac{2\pi^{d/2}}{\Gamma (d/2)}r^{d-1}.$$

Now using the change of variables *u* = |*x* − *rξ*|^2^/4*ks* on the integral appearing in the right-hand side of (2.18) yields:

2.19 $$\begin{array}{rl} \int_{0}^{t}\;\text{d}s[s^{-d/2}\;{\text{e}}^{-|x-r\xi {|}^{2}/4ks}] & =\int_{+\infty }^{|x-r\xi {|}^{2}/4kt}\;\text{d}u\frac{{\left(4ku\right)}^{d/2}}{|x-r\xi {|}^{d}}\left(\frac{-|x-r\xi {|}^{2}}{4ku^{2}}\right){\text{e}}^{-u}\\ & =\frac{{\left(4k\right)}^{d/2-1}}{|x-r\xi {|}^{d-2}}\int_{|x-r\xi {|}^{2}/4kt}^{+\infty }\;\text{d}u[u^{d/2-1-1}\;{\text{e}}^{-u}]\\ & =\frac{{\left(4k\right)}^{d/2-1}}{|x-r\xi {|}^{d-2}}\Gamma \left(\frac{d}{2}-1,\frac{|x-r\xi {|}^{2}}{4kt}\right), \end{array}$$

where the upper incomplete Gamma function Γ(d/2−1,⋅) is defined for all *y* > 0 by:

2.20 $$\Gamma \left(\frac{d}{2}-1,y\right)=\int_{y}^{+\infty }\;\text{d}u[u^{d/2-1-1}\;{\text{e}}^{-u}].$$

In our case *y* = |*x* − *rξ*|^*d*−2^/(4*kt*) → +∞ as *r* → +∞. Thus, we need an equivalent of Γ(d/2−1,y) as *y* → +∞. To this aim, we do an integration by parts on (2.20) to get:

2.21 $$\Gamma \left(\frac{d}{2}-1,y\right)={\text{e}}^{-y}y^{d/2-2}+(d/2-2)\int_{y}^{+\infty }\;\text{d}u[u^{d/2-1-1}u^{-1}\;{\text{e}}^{-u}].$$

Then, as *u* ≥ *y*, one observes that

$$\left|\int_{y}^{+\infty }\;\text{d}u[u^{d/2-1-1}u^{-1}\;{\text{e}}^{-u}]\right|\leq \frac{1}{y}\Gamma \left(\frac{d}{2}-1,y\right)$$

and thus concludes from (2.21) that:

2.22 $$\Gamma \left(\frac{d}{2}-1,y\right)={\text{e}}^{-y}y^{d/2-2}(1+o(1)),\;\text{as}\;y\to +\infty .$$

Combining (2.18), (2.20) and the equivalent of the incomplete Gamma function (2.22) for *y* = |*x* − *rξ*|^*d*−2^/(4*kt*) gives that for *r* large enough:

2.23 $$\begin{array}{rl} |I_{1}(x,t;r)| & \leq 2C\;r^{m}\;{\text{e}}^{ar^{b}}A_{d}(r)\frac{{\left(4k\right)}^{d/2-1}}{|x-r\xi {|}^{d-2}}{\left(\frac{|x-r\xi |}{4kt}\right)}^{d/2-2}\;{\text{e}}^{-|x-r\xi {|}^{2}/4kt}\\ & \leq {\tilde{C}}_{x,t}\;r^{m+d/2-1}\;{\text{e}}^{-|x-r\xi {|}^{2}/4kt+ar^{b}}, \end{array}$$

where ${\tilde{C}}_{x,t}$ is a positive constant that depends only *x* and *t*, which are fixed here. To conclude, observe that the upper bound in (2.23) goes to 0 as *r* → ∞ since *b* < 2. This statement holds for any *x* outside of $\bar{\Omega }$ and any *t* > 0, yielding the representation (2.13). ▪

**Remark 2.7.**

We point out that the regularity assumption $v\in C^{\left(2,1\right)}((R^{d}-\Omega )\times [0,+\infty ))$ of the solution in theorem 2.6 can be relaxed, as long as the duality pairings in the representation of the solution (2.7) are integrals. Indeed, the proof of theorem 2.6 still holds with weaker regularity assumptions on *v* but for a bounded open set Ω with a *C*^2^ smooth boundary ∂Ω (see [41,53]), which is slightly more than the Lipschitz regularity imposed on ∂Ω in theorem 2.6 for *C*^(2,1)^ solutions. Indeed, if ∂Ω is *C*^2^, our proof works under a Sobolev local regularity, namely if the solution *v* of the heat equation (2.1) (in the sense of distributions with zero initial conditions and a source term spatially supported in the compact set $\bar{\Omega }$) belongs to $H^{2\alpha ,\alpha }(O\times (0,T))$ for any *T* > 0, any open bounded set $O\subset R^{d}-\bar{\Omega }$ and some fixed Sobolev index *α* > 3/4. This assumption and the zero initial condition of *v* allows us to apply the representation formula (2.7) (instead of (2.14)) in the proof for $O=B(0,r)-\bar{\Omega }$. The representation (2.7) involves duality pairings between Sobolev spaces of the boundary ∂O×(0,t) with $\partial O=\partial \Omega \cup S^{d}(0,r)$. However, by the trace theorem 2.1 p. 9 in [53], the assumed local Sobolev regularity ensures that ∂*v*/∂*n* belongs to $L^{2}(\partial \Omega \times (0,t))$ and to *L*^2^(*S*^*d*^(0, *r*) × (0, *t*)) for *t* > 0 and *r* sufficiently large. Thus, the integrals *I*_1_(*x*, *t*;*r*) and *I*_2_(*x*, *t*;*r*) can be interpreted not only as duality pairings but as integrals, as in (2.14). Furthermore, by interior regularity (and even hypoellipticity) of the differential operator in the heat equation (see [37] theorem 1.1 p. 192), one has $v\in C^{\infty }(R^{d}-\bar{\Omega })\times (0,+\infty )$. Thus, as *v* is smooth on this set, the growth condition 2.4 is still well defined. Therefore, the proof of theorem 2.6 follows exactly in the same way and yields the representation formula (2.13) in this new setting.

The heat kernel and its spatial derivatives (which all solve the heat equation) satisfy the growth condition 2.4, as we see next.

**Lemma 2.8.**

*In dimension* *d* ≥ 1, *the heat kernel and all its spatial derivatives satisfy the growth condition* (2.12) *for some* *C* > 0 *and any* *a* ≥ 0, *b* ∈ [0, 2), *r*_0_ > 1 *and non-negative integer* *m*.

**Proof.**

We first introduce the notation $\partial_{x}^{\alpha }\psi$ for arbitrary spatial derivatives of a smooth function (x,t)↦ψ(x,t) on $R^{d}\times (0,\infty )$:

$$\partial_{x}^{\alpha }\psi (x,t)=\partial_{x_{1}}^{\alpha_{1}}\;\partial_{x_{2}}^{\alpha_{2}}\ldots \;\partial_{x_{d}}^{\alpha_{d}}\psi (x_{1},x_{2},\ldots ,x_{d},t),\;\text{where}\;x=(x_{1},x_{2},\ldots ,x_{d}).$$

Here, *α* = (*α*_1_, …, *α*_*d*_) is a multi-index, where *α*_*i*_ is the order of differentiation in *x*_*i*_.

Since the heat kernel *K* is in $C^{\infty }(R^{d}\times (0,+\infty ))$, an induction on the degree of differentiation reveals that for $\left(x,t)\in R^{d}\times (0,+\infty \right)$, *K* and any of its spatial derivatives have the form,

2.24 $$\partial_{x}^{\alpha }K(x,t)=P(x_{1}/t,x_{2}/t,\ldots ,x_{d}/t,1/t)\;K(x,t),$$

where *P* is a multi-variate polynomial. In the particular case *α* = (0, 0, …, 0), we have $\partial_{\alpha }K=K$ and thus *P* = 1. Using the triangle inequality on the expression (2.24) of $\partial_{x}^{\alpha }K$ leads to:

2.25 $$|\partial_{x}^{\alpha }K(x,t)|\leq Q(|x|/t,|x|/t,\ldots ,|x|/t,1/t)\;|K(x,t)|\;\text{for}\;(x,t)\in R^{d}\times (0,\infty ),$$

where *Q* is a polynomial that has the same monomial terms as *P*, but whose coefficients are given by the modulus of the coefficients of *P*.

Let *r*_0_ ≥ 1 and |*x*| = *r* > *r*_0_. We set *u* = |*x*|^2^/*t* in the right-hand side of (2.25). As |*x*|/*t* ≤ |*x*|^2^/*t* = *u* (since 1 ≤ |*x*| ≤ |*x*|^2^), $1/t=u/|x{|}^{2}\leq u/r_{0}^{2}$ (since |*x*| > *r*_0_) and the coefficients of *Q* are positive, it follows from (2.25) and the expression (2.3) of *K* that:

2.26 $$|\partial_{x}^{\alpha }K(x,t)|\leq Q(u,u,\ldots ,u,u/r_{0}^{2})\;{\left(4\;k\pi \right)}^{-d/2}{\left(u/r_{0}^{2}\right)}^{d/2}\;{\text{e}}^{-u/(4k)}\;\text{for}\;|x|>r_{0}\;\text{and}\;t>0.$$

As *Q* is a polynomial, due to the exponential term, the right-hand side of (2.26) is clearly bounded for *u* > 0, thus there is a constant *C*_1,*α*_ > 0 (depending only on *α*, *d* and *r*_0_) such that:

2.27 $$|\partial_{x}^{\alpha }K(x,t)|\leq C_{1,\alpha }\;\text{for}\;|x|>r_{0}\;\text{and}\;t>0.$$

Now, as the bound (2.26) holds for any *α*, one immediately deduces that there is a *C*_2,*α*_ such that

2.28 $$|\nabla (\partial_{x}^{\alpha }K(r\xi ,t))\cdot n|\leq |\nabla (\partial_{x}^{\alpha }K(r\xi ,t))|\leq C_{2,\alpha }\;\text{ for}x=r\xi ,\xi \in S^{d}(0,1),r>r_{0}\text{and}t>0.$$

Thus, by (2.28), (2.26) and the bound *r*/*t* ≤ *r*^2^/*t* = *u* (as *r* > *r*_0_ ≥ 1), there is a *C*_3,*α*_ > 0 such that:

2.29 $$\begin{array}{rl} \left|\nabla (\partial_{\alpha }K(r\xi ,t))\cdot n+\frac{r}{2kt}\partial_{\alpha }K(r\xi ,t)\right| & \leq |\nabla (\partial_{\alpha }K(r\xi ,t))\cdot n|+\frac{u}{2k}|\partial_{\alpha }K(r\xi ,t)|\\ & \leq C_{2,\alpha }+\frac{{\left(2k\right)}^{-1}}{{\left(4\pi kr_{0}^{2}\right)}^{d/2}}\;Q\left(u,u,\ldots ,u,\frac{u}{r_{0}^{2}}\right)u^{d/2+1}\;{\text{e}}^{-u/(4k)}\\ & \leq C_{3,\alpha }, \end{array}$$

for any *r* > *r*_0_, *ξ* ∈ *S*^*d*^(0, 1) and *t* > 0. ▪

**Remark 2.9.**

As in remark 2.3 for bounded Ω, the representation formula (2.13) satisfies some stability due to the maximum principle, see [44,46]. However, as $R^{d}-\bar{\Omega }$ is unbounded, it requires a bound that controls the growth of the functions when |*x*| → +∞, namely, one assumes that there exist *A*, *a* > 0 such that:

2.30 $$|u(x,t)|\leq A\;{\text{e}}^{a|x{|}^{2}},\text{ for }(x,t)\in (R^{d}-\bar{\Omega })\times (0,T],$$

for some finite *T* > 0. This last condition allows Gaussian growth and is similar to condition 2.4.

More precisely, let $v_{j}\in C^{0}((R^{d}-\Omega )\times [0,T])$ for *j* = 1, 2 be two solutions of the homogeneous heat equation in $\left(R^{d}-\bar{\Omega })\times (0,T\right)$ in the distributional sense that satisfy the growth condition (2.30). Again by hypoellipticity (see [37] theorem 1.1 p. 192), *u*_*j*_ is indeed a smooth solution of the homogeneous heat equation on $\left(R^{d}-\bar{\Omega })\times (0,T\right)$ for *j* = 1, 2 since it is *C*^∞^ on this set. Then by the maximum principle (e.g. [46], ch. 3, §3, theorem 6) one has:

2.31 $$\underset{\left(R^{d}-\Omega )\times [0,T\right]}{\sup}|v_{2}(x,t)-v_{1}(x,t)|=\underset{\left((R^{d}-\Omega )\times \{0\})\cup (\partial \Omega \times [0,T]\right)}{\sup}|v_{2}(x,t)-v_{1}(x,t)|.$$

Note that the proof of ([46], ch. 3, §3, theorem 6) is done on all of $R^{d}$, but can be adapted to $R^{d}-\bar{\Omega }$ with Ω a bounded. Furthermore, if the initial condition is harmonic and decays to 0 as |*x*| → +∞, using the maximum principle for the Laplace equation in unbounded domains, one can simplify (2.31) to include only surface terms in the right-hand side

2.32 $$\max_{\left(R^{d}-\Omega )\times [0,T\right]}|v_{2}(x,t)-v_{1}(x,t)|=\max_{\partial \Omega \times [0,T]}|v_{2}(x,t)-v_{1}(x,t)|.$$

Thus, in the space of solutions of the homogeneous heat equation (that satisfy (2.30) and the regularity described above), both (2.31) and (2.32) tell us that an error committed on the initial condition or on the Dirichlet boundary data controls the reconstruction error of $v_{\Omega }$ on $\left(R^{d}-\bar{\Omega })\times (0,T\right]$, in the supremum norm. Furthermore, uniqueness on $\left(R^{d}-\Omega )\times [0,T\right]$ for the heat equation exterior Dirichlet problem follows from the maximum principle equality (2.31), provided the growth condition (2.30) and regularity assumptions hold. Moreover if (2.30) is satisfied for any *t* > 0, this uniqueness result extends to $\left(R^{d}-\Omega )\times [0,+\infty \right)$, assuming the same regularity assumptions but with an infinite time.

Finally, as in remark 2.3, under the additional assumption that the open set $R^{d}-\bar{\Omega }$ is connected, if a maximum is attained in (2.31) at $\left(x_{0},t_{0})\in (R^{d}-\bar{\Omega })\times (0,T\right]$ then there exists a real constant *C* such that *v*_2_(*x*, *t*) − *v*_1_(*x*, *t*) = *C* on $\left(R^{d}-\Omega )\times [0,t_{0}\right]$. Furthermore, one shows that if one considers the formula (2.32), this last property holds also for *t*_0_ = 0 and the constant *C* has to be zero (since in formula (2.32), one assumes that the initial conditions decay to 0 when |*x*| → +∞ which imposes that *C* = 0).

**Figure 5.**
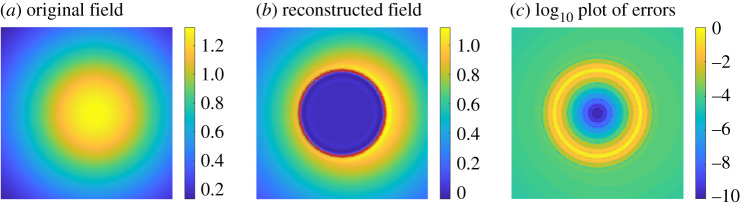
Numerical example of the exterior field reproduction problem. The details of this example are the same as figure 3, but with the point source moved to (0.5, 0.55). We generated a plot similar to (*c*) by taking the maximum over the time interval [0.2, 0.3] for each grid point (the plot being very similar to (*c*), we include code to generate it in [42]). This indicates that the maximum error is attained near ∂Ω in space (and in time at *t* = 0.2), conforming to the maximum principle (remarks 2.3, 2.9). We can use here the maximum principle on the computed error because the numerical methods we use generates solutions to the heat equation (see §5.). (*a*) Original field, (*b*) reproduced field, (*c*) log _10_ plot of errors. (Online version in colour.)

A numerical example of the exterior reproduction of a field can be seen in figure 5. The details of the example are the same as those in figure 3, except the point source has been moved to (0.5, 0.55).

### (c) Numerical sensitivity study of field reproduction

We study the sensitivity of the numerical approximation of the boundary representation formulae (2.5) and (2.13) to the following factors: (a) the spatial discretization (number of points on ∂Ω), (b) the temporal discretization (number of time steps) and (c) errors in the densities appearing in the boundary representation formulae. As can be seen in figures 3*c* and 5*c*, the reproduction error peaks close to the boundary, so we decided to exclude a neighbourhood of ∂Ω from the error measures we present. The numerical approximation of the boundary reproduction formulae is explained in detail in §5. Here, we keep the same domain Ω as in the examples of §§2.a and 2.b. The boundary integral representations were used to approximate the field on a 100 × 100 uniform grid of [0, 1]^2^ and the thermal diffusivity was taken to be *k* = 0.2.

The first case we consider is that of the interior reproduction problem, i.e. when the source distribution is spatially supported in $R^{2}-\Omega$. We expect using (2.5) that $u_{\Omega }=u$ inside Ω and $u_{\Omega }=0$ outside $\bar{\Omega }$. To evaluate the quality of the numerical approximation we make, we calculate the relative reproduction error on a slightly smaller domain $\Omega_{-s}=B(x_{0},(1-s)r)$,

$${\text{relerr}}_{-}(u;t)=\frac{||u(\cdot ,t)-u_{\Omega }(\cdot ,t)|{|}_{L^{2}(\Omega_{-s})}}{||u(\cdot ,t)|{|}_{L^{2}(\Omega_{-s})}},$$

where we used the *L*^2^ norm of a function over some set *R*, namely

$$||f|{|}_{L^{2}(R)}={\left(\int_{R}|f(x){|}^{2}\;\text{d}x\right)}^{1/2}.$$

We also calculate the absolute error outside of a slightly larger domain $\Omega_{s}=B(x_{0},(1+s)r)$, i.e.

$${\text{err}}_{+}(u;t)=||u_{\Omega }(\cdot ,t)|{|}_{L^{2}({\left[0,1\right]}^{2}-\Omega_{s})}.$$

**Figure 6.**
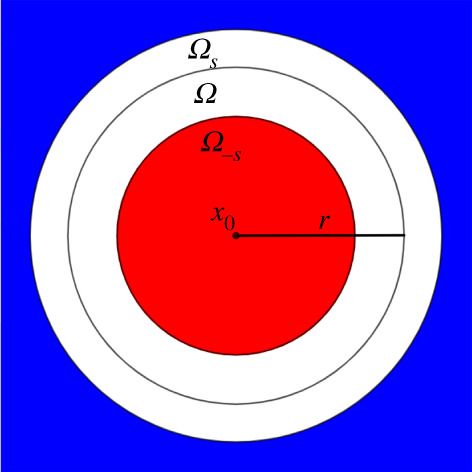
We evaluate the field reproduction errors on regions that exclude a neighbourhood of the boundary ∂Ω. For thenumerical experiments, we took $\Omega =B(x_{0},r)\subset {\left[0,1\right]}^{2}$. The interior reproduction error is evaluated on $\Omega_{-s}=B(x_{0},(1-s)r)$ (in red) while the exterior one is evaluated on ${\left[0,1\right]}^{2}-\Omega_{s}$ (in blue), where $\Omega_{s}=B(x_{0},(1+s)r)$. (Online version in colour.)

The *L*^2^ norms appearing in the error quantities that we consider are approximated using Riemann sums on the 100 × 100 grid of [0, 1]^2^. The domains of interest, ${\left[0,1\right]}^{2}-\Omega_{s}$ and $\Omega_{-s}$, are illustrated by the blue and red regions of figure 6, respectively. In our numerical experiments, we chose *s* = 0.05 to get a buffer annulus at ±5% of *r*. The field *u* is generated by a point source *δ*(*x* − *x*_0_, *t*) located at *x* = *x*_0_ and *t* = 0.

Similarly for the exterior reproduction problem, where we want to reproduce a field *v* satisfying the heat equation with a source term spatially supported in Ω and satisfying the radiation-type boundary condition (2.12), we calculate the absolute interior error

$${\text{err}}_{-}(v;t)=||v_{\Omega }(\cdot ,t)|{|}_{L^{2}(\Omega_{-s})}$$

and the relative exterior error

$${\text{relerr}}_{+}(v;t)=\frac{||v(\cdot ,t)-v_{\Omega }(\cdot ,t)|{|}_{L^{2}({\left[0,1\right]}^{2}-\Omega_{s})}}{||v(\cdot ,t)|{|}_{L^{2}({\left[0,1\right]}^{2}-\Omega_{s})}}.$$

For the exterior reproduction studies, the field *u* is produced by a delta source located at *x*_0_ = (0.5, 0.55) and *t* = 0. Some one-dimensional numerical examples are provided in electronic supplementary material, appendix A.

#### (i) Sensitivity to spatial discretization

**Figure 7.**
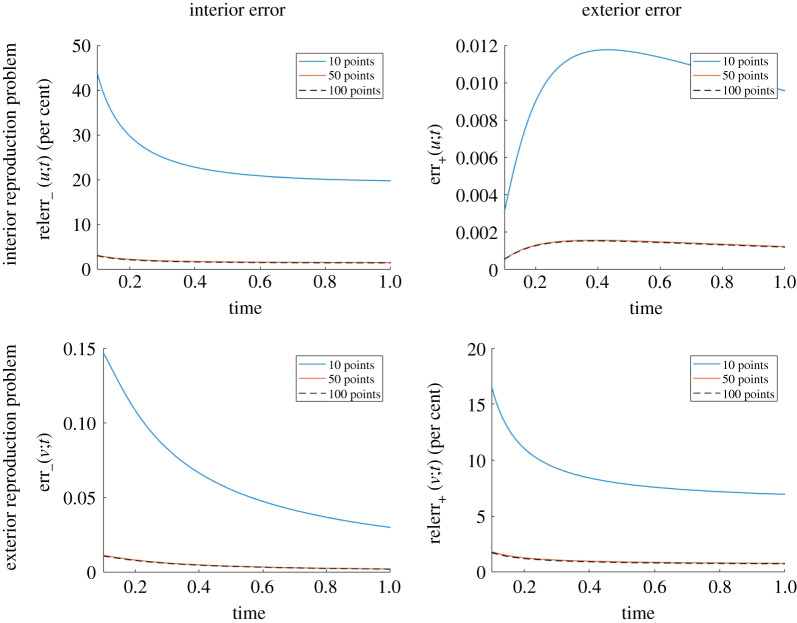
Influence of the number of points used to discretize ∂Ω on the reproduction error for the interior reproduction problem of a point source located at *x*_0_ = (0, 0), *t* = 0 (top row) and for the exterior reproduction problem of a point source located at *x*_0_ = (0.5, 0.55), *t* = 0 (bottom row), both with thermal diffusivity *k* = 0.2. Here, ∂Ω is the circle of radius 0.25 centred at (0.5, 0.5). Since the errors for small times are large, we only show the errors for *t* ≥ 0.1. (Online version in colour.)

In figure 7, we illustrate the changes in reproduction error for both the interior (figure 7, first row) and exterior (figure 7, second row) reproduction problems. For both studies, a uniform discretization of ∂Ω is used and 1000 uniform time steps. While increasing the number of points on ∂Ω decreases the error in all cases, the decrease from 50 to 100 points is modest. We think this is due to the temporal discretization error being dominant.

#### (ii) Sensitivity to temporal discretization

**Figure 8.**
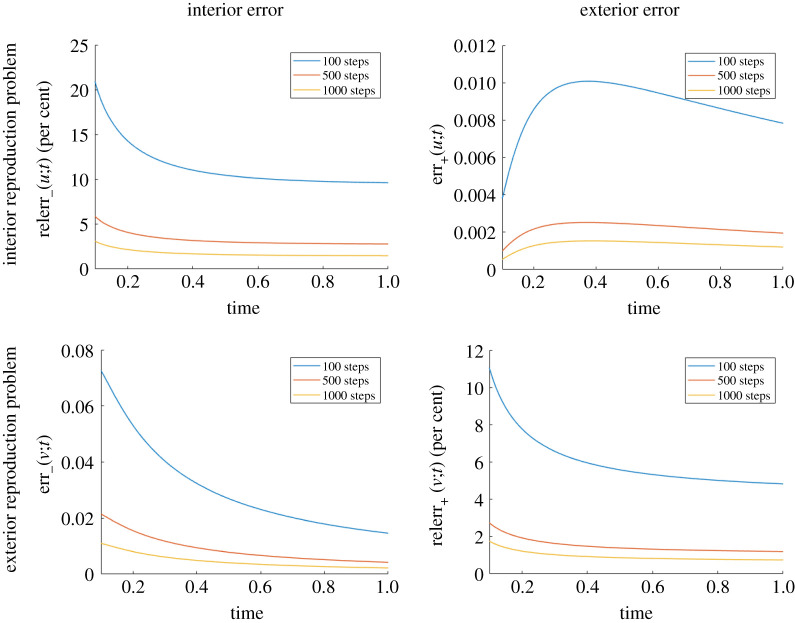
Influence of the number of time steps on the reproduction error for the interior reproduction problem of a point source located at *x*_0_ = (0, 0), *t* = 0 (first row) and for the exterior reproduction problem of a point source located at *x*_0_ = (0.5, 0.55), *t* = 0 (second row), both with thermal diffusivity *k* = 0.2. Here, ∂Ω is the circle of radius 0.25 centred at (0.5, 0.5). Since the errors for small times are large, we only show the errors for *t* ≥ 0.1. (Online version in colour.)

We report in figure 8 the change in reproduction error as we increase the number of time steps while keeping the number of uniformly spaced points used to discretize ∂Ω fixed and equal to 100. This is done for both the interior (figure 8, first row) and exterior (figure 8, second row) reproduction problems. For a fixed time, the errors decrease with the number of time steps, as expected.

#### (iii) Sensitivity to errors in the densities

**Figure 9.**
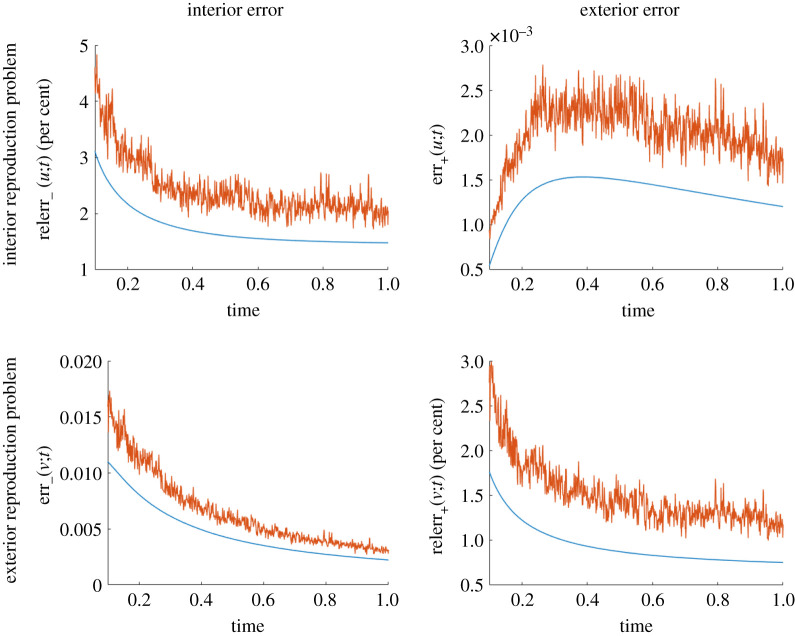
Influence of random perturbations added to the monopole and dipole densities on the reproduction error for the interior reproductionproblem of a point source located at *x*_0_ = (0, 0), *t* = 0 (first row) and for the exterior reproduction problem of a point source located at *x*_0_ = (0.5, 0.55), *t* = 0 (second row), both with thermal diffusivity *k* = 0.2. Here, ∂Ω is the circle of radius 0.25 centred at (0.5, 0.5). Since the errors for small times are large, we only show the errors for *t* ≥ 0.1. In orange: error with random perturbations. In blue: error obtained with the unperturbed densities. (Online version in colour.)

In practice it cannot be assumed that the field to be reproduced is perfectly known so we report in figure 9 how the reproduction error is affected by errors in the monopole and dipole densities appearing in (2.5) and (2.13) when discretized with 1000 time steps and 100 points on ∂Ω.

Say $\phi^{\left(n\right)}\in R^{100}$ is a vector representing the values of either the monopole or dipole density in one of the boundary representation formulae at time *nδt*, where *δt* is the time step. We perturb *ϕ*^(*n*)^ with a vector $\delta \phi^{\left(n\right)}\in R^{100}$ with independent identically distributed zero mean Gaussian entries with standard deviation being a fraction (3%) of $||\phi^{\left(n\right)}|{|}_{2}$. For clarity, we only show the error for a single realization of the perturbation *δϕ*^(*n*)^. The errors we observe in figure 9 oscillate rapidly because we introduced a random perturbation at every single time step. As expected, the error we introduced in the densities increases the overall error at every single time step. We include code to generate the spatial distribution of the reconstruction errors in [42]. We observe that the maximum error over a time window is attained near ∂Ω as predicted by the maximum principle (remarks 2.3, 2.9). Since the additive noise comes from perturbing the monopole and dipole densities, this perturbation is a smooth solution of the heat equation (see §5.), which makes the maximum principle applicable.

**Remark 2.10.**

In the numerical results we present, the exterior errors, relative and absolute, are smaller than the interior errors. We also see a transient effect in the absolute exterior error. We believe this is similar to the temperature distribution near a point source, which also increases and then decreases with time. Since the errors that we report in figures 7–9 are based on the *L*^2^ norm, the maximum principle considerations of remarks 2.3, 2.9 do not apply directly to these numerical experiments.

## 3. Cloaking

The goal here is to use the results from §2. to cloak sources or objects inside a cloaked region, by placing sources on the surface of the region. By cloaking we mean that it is hard to detect the object or source from only thermal measurements made outside the cloaked region. The boundary representation formulae of §2. give us the appropriate surface source distribution. We start in §3.a with the interior cloaking of a source, directly applying the boundary representation formula in §2.b. The interior cloaking of an object is illustrated in §3.b by using the boundary representation formula in §2.a. The boundary representation formulae impose restrictions on what can be cloaked and how. In either case, the field must be known for all time and with no sources in the region where it is reproduced. For the interior cloaking of a source, the temperature field generated by this source must also satisfy condition 2.4.

### (a) Cloaking a source in an unbounded domain

Given certain kinds of localized heat source distributions, we can find an active surface surrounding the source so that the source cannot be detected by an observer outside the surface. Let *v*_*i*_(*x*, *t*) be a free space solution to the heat equation (2.2) with zero initial condition and compactly supported source distribution *h*(*x*, *t*). Let Ω be an open bounded set (with Lipschitz boundary) that contains the support of the source *h*(*x*, *t*) for *t* > 0. In an analogy with wave problems, we call *v*_*i*_ the ‘incident field’ and we further assume that it satisfies the growth condition 2.4. By theorem 2.6, we can find monopole and dipole densities on ∂Ω so that the boundary representation formula (2.13) gives −*v*_*i*_ outside of $\bar{\Omega }$ and 0 inside Ω. We call this the cloaking field *v*_*c*_ and it is given for *t* > 0 by

3.1 $$v_{c}(x,t)=\left\{ \begin{array}{ll} 0 & x\in \Omega \\ -v_{i}(x,t) & x\notin \bar{\Omega }. \end{array}\right.$$

In this manner, the total field *v*_tot_ = *v*_*i*_ + *v*_*c*_ is zero outside of $\bar{\Omega }$ and equal to *v*_*i*_ inside Ω. Because the active surface ∂Ω perfectly cancels the effect of the source *h*(*x*, *t*) for $x\notin \bar{\Omega }$, the source cannot be detected by an observer. Figure 5 shows a numerical example of *v*_*c*_.

### (b) Cloaking passive objects in an unbounded domain

One way to detect an object in free space using only thermal measurements would be to generate an incident or probing field *u*_*i*_(*x*, *t*) with a source distribution *h*(*x*, *t*), i.e. a solution to the heat equation (2.2) in free space with zero initial condition and *h* as its source term. In the presence of an object, the total field is given by *u*_tot_ = *u*_*i*_ + *u*_*s*_, where *u*_*s*_ is the field ‘scattered’ by the object, borrowing terminology from the wave equation. The scattered field is produced by the interaction between the incident field and the object and depends on the properties of the object (boundary condition, heat conductivity, etc.). We point out that *u*_*s*_(*x*, 0) = 0 because *u*_tot_(*x*, 0) = *u*_*i*_(*x*, 0). Having *u*_*s*_ ≠ 0 reveals the presence of an object. In the following, we assume that the object is ‘passive’, meaning that the scattered field is linear in the incident field. In particular, this means that *u*_*s*_ = 0 when *u*_*i*_ = 0. Examples of passive objects include objects with homogeneous linear boundary conditions (e.g. Dirichlet, Neumann or Robin) or objects with a heat conductivity that is different from that of the surrounding medium (see e.g. [54,55] for transmission problems for the heat equation). We point out that the object is assumed to be open with Lipschitz boundary.

The results in §2. can be used to cloak a passive object *R* by placing it inside a cloaking region Ω (i.e. a bounded open set Ω with smooth boundary such that $\bar{R}\subset \Omega$) and makes this whole region invisible from probing incident fields *u*_*i*_ generated by a source *h* spatially supported in $R^{d}-\Omega$. Indeed, by controlling monopoles and dipoles on ∂Ω, the region Ω and the object within can be made indistinguishable from a patch of homogeneous medium, from the perspective of thermal measurements outside of $\bar{\Omega }$. In a similar manner to §3.a, the idea is to use (2.5) to cancel the incident field in Ω, while leaving the outside of $\bar{\Omega }$ unperturbed. The cloaking field, *u*_*c*_, produced by this active surface ∂Ω is then for *t* > 0:

3.2 $$u_{c}(x,t)=\left\{ \begin{array}{ll} -u_{i}(x,t) & x\in \Omega ,\\ 0 & x\notin \bar{\Omega }. \end{array}\right.$$

In principle, this cloaking field can be used to perfectly cancel the incident field in Ω for all *t* > 0. Since the temperature of the ‘modified incident field’: *u*_*i*_ + *u*_*c*_ is zero in Ω, the temperature field surrounding the object vanishes and no scattered field is produced. In practice, the field *u*_*i*_ + *u*_*c*_ in the vicinity of the object does not perfectly vanish, but we expect it to be sufficiently close to zero so that the scattered field *u*_*s*_ is very small (because of linearity).

**Figure 10.**
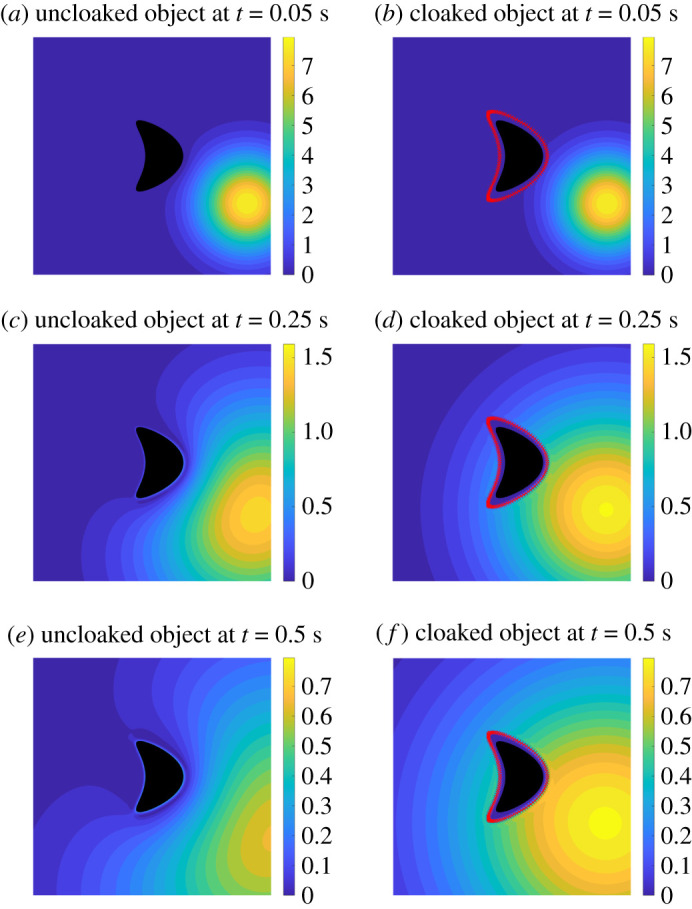
Numerical example of the object cloaking problem, with object to hide having homogeneous Dirichlet boundary conditions. The incident field is produced by a point source at *x* = (0.9, 0.3) and *t* = 0 s. The images on the left show snapshots of the object without the cloak at (*a*) *t* = 0.05 s, (*c*) *t* = 0.25 s and (*e*) *t* = 0.5 s. The images on the right show the corresponding snapshots when the cloak is active at (*b*) *t* = 0.05 s, (*d*) *t* = 0.25 s and (*f* ) *t* = 0.5 s. Placing the cloak close to the object is a challenging simulation because we expect that errors will be higher near the boundary as in figure 3. (See also the movie in the electronic supplementary material). (Online version in colour.)

Our technique is illustrated with an object with homogeneous Dirichlet boundary conditions in figure 10. Here, the field *u*_*i*_ is generated by a point source at *x* = (0.9, 0.3) and *t* = 0. For the heat equation, we took *k* = 0.2 and the cloaked region is $\Omega =B(x_{0},r)$ with *x*_0_ = (0.5, 0.5) and *r* = 1/3. We computed the fields on the unit square [0, 1]^2^ with a 200 × 200 uniform grid. The field *u*_*c*_ is found by approximating the integral (2.5) using the midpoint rule in time with 600 equal length subintervals of [0, 0.5] and the trapezoidal rule on ∂Ω with 128 uniformly spaced points. A more detailed explanation, including how the scattered fields are calculated, is in §5. We represent in figure 10 the total fields respectively generated by the incident field *u*_*i*_ (left column) and by the ‘modified incident field’: *u*_*i*_ + *u*_*c*_ (right column). As can be seen in the right column, the temperature fields, outside of the cloaked region, are indistinguishable from the incident field *u*_*i*_. We do not include a detailed error plot for this configuration, as the error is similar to the one we encountered when studying the interior reproduction problem.

**Remark 3.1.**

Here are two ways of dealing with *active objects*, i.e. that are not passive. First, if the object produces a non-zero scattered field *u*_*s*_ when *u*_*i*_ = 0, the object acts as a source and *u*_*s*_ can be cancelled using the technique in §3.a. This presumes perfect knowledge of *u*_*s*_ and *u*_*i*_. Second, if the object does not produce a scattered field when immersed in some harmonic field *u*_0_, we can use as a cloaking field *u*_*c*_(*x*, *t*) = −*u*_*i*_(*x*, *t*) + *u*_0_(*x*) for x∈Ω and *u*_*c*_(*x*, *t*) = 0 for $x\notin \bar{\Omega }$, instead of (3.2). An example of such an object would be one with a constant *c* ≠ 0 Dirichlet boundary condition. By our assumption, the field *u*_0_(*x*) = *c* does not create any scattering, regardless of the shape of the object.

## 4. Mimicking

Another possible application of the boundary representation formulae (2.5) and (2.13) is to mimic sources or passive objects. This is done in two steps. First, we cancel out the original source or suppress the scattering of the original object using a source distribution on a surface ∂Ω surrounding the object. Second, we adjust the source distribution so that the object or source appears to the observer as another object or source. We illustrate this idea with two cases: making sources look like other sources (§4.a) and making a passive object look like a different passive object (§4.b). Other combinations are possible but are not presented here.

### (a) Source mimicking

For source mimicking, we consider the problem where there is a compactly supported source distribution, *f*(*x*, *t*), which we seek to make appear as a different compactly supported source distribution, *g*(*x*, *t*), from thermal measurements outside of a region $\bar{\Omega }$ (where Ω is an open bounded set with Lipschitz boundary). The support of both distributions is assumed to be contained in $\bar{\Omega }$ for all *t* > 0. The two corresponding solutions to the heat equation (2.2) are *v*(*x*, *t*;*f*) and *v*(*x*, *t*;*g*), and we further assume they satisfy condition 2.4.

Mimicking can be achieved by simultaneously cancelling the field *v*(*x*, *t*;*f*) outside $\bar{\Omega }$ and adding *v*(*x*, *t*;*g*), also outside $\bar{\Omega }$. Both can be done using the results in §3.a. That is, using (3.1) we can find a monopole and dipole density on ∂Ω that generates a field

4.1 $$v_{c}(x,t)=\left\{ \begin{array}{ll} 0 & x\in \Omega ,\\ v(x,t;g)-v(x,t;f) & x\notin \bar{\Omega }. \end{array}\right.$$

In this way, the field *v*(*x*, *t*;*f*) + *v*_*c*_(*x*, *t*) is equal to *v*(*x*, *t*;*g*) outside of $\bar{\Omega }$, as desired.

**Figure 11.**
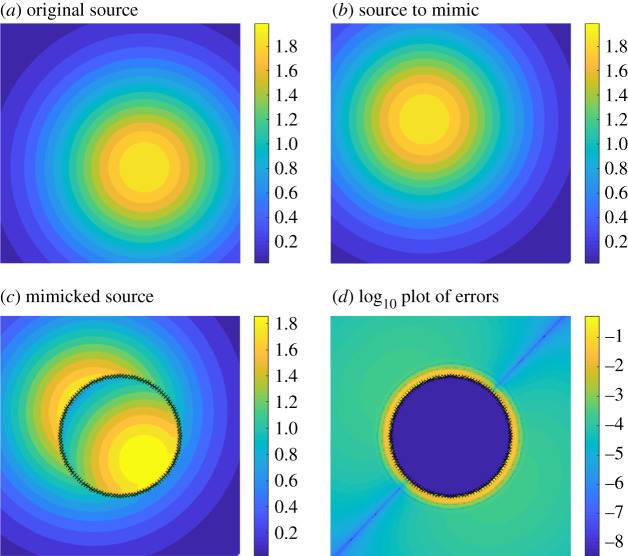
Time snapshots from a numerical example of the source mimicking problem with point sources at *t* = 0.2 s. In (*c*), the original point source in (*a*) is made to appear as the point source in (*b*) from the perspective outside of the cloaking region Ω. The point source in (*a*) is at *x* = (0.6, 0.4) and *t* = 0 and in (*b*) is at *x* = (0.39, 0.6) and *t* = 0. A plot of the log _10_ errors in the exterior appears in (*d*). The diagonal line is an artefact due to the symmetry of the problem. A similar problem with point sources at *x* = (0.5, 0.4) and *x* = (0.5, 0.6) produces a horizontal line. (Online version in colour.)

A numerical example to illustrate the method is given in figure 11. Fields are calculated in [0, 1]^2^ using a uniform grid of 200 by 200 points at *t* = 0.2 s. Here, a point source at *y*^(1)^ = (0.6, 0.4) and *t* = 0 is made to appear as a point source at *y*^(2)^ = (0.39, 0.6) and *t* = 0, from thermal measurements outside of $\bar{\Omega }$. Figure 11*a*,*b* represent the fields *v*(*x*, *t*;*f*) and *v*(*x*, *t*;*g*), where *f*(*x*, *t*) = *δ*(*x* − *y*^(1)^, *t*) and *g*(*x*, *t*) = *δ*(*x* − *y*^(2)^, *t*). Figure 11*c* shows the field *v*(*x*, *t*;*f*) + *v*_*c*_(*x*, *t*), where *v*_*c*_ has been constructed by applying (4.1). An error plot is shown in figure 11*d*, where the error is largest near the boundary, as we expect based on the sensitivity analysis of §2. We believe the diagonal line with small error in figure 11*d* is an artefact of our choice of sources.

### (b) Mimicking a passive object

Consider a passive object *R* that is completely contained in an open set Ω and a source exterior to Ω that produces the incident field *u*_*i*_. The goal is to make *R* look like a different passive object, *S*, from thermal measurements outside $\bar{\Omega }$. To achieve this, we can use linearity and both the interior and exterior boundary representation formulae to find monopole and dipole densities on ∂Ω producing a field

4.2 $$u_{c}(x,t)=\left\{ \begin{array}{ll} -u_{i}(x,t), & x\in \Omega ,\\ v_{s}(x,t), & x\notin \bar{\Omega }, \end{array}\right.$$

where *v*_*s*_(*x*, *t*) is the scattered field corresponding to the object *S*, included also in Ω, resulting from the incident field *u*_*i*_(*x*, *t*). In this fashion, the total field is *u*_*i*_(*x*, *t*) + *v*_*s*_(*x*, *t*) outside of $\bar{\Omega }$ and 0 inside of Ω. Its associated scattered field is *v*_*s*_(*x*, *t*) outside of $\bar{\Omega }$ and 0 inside of Ω−R as desired.

**Figure 12.**
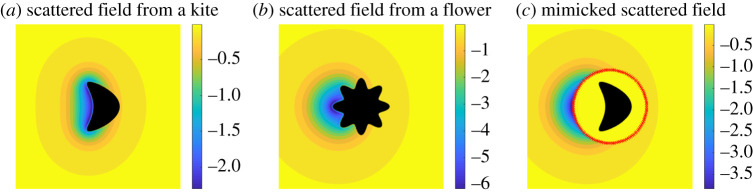
Numerical example of the object mimicking problem with a ‘kite’ and ‘flower’ object, both with homogeneous Dirichlet boundary conditions. A snapshot of the scattered field from the original object at time *t* = 0.05 s appears in (*a*). In (*c*), the scattered field from the original object is made to appear as the scattered field from the object in (*b*) from the perspective of thermal measurements outside of the cloaking region $\bar{\Omega }$. (*a*) Scattered field from a kite, (*b*) scattered field from a flower and (*c*) mimicked scattered field. (Online version in colour.)

To illustrate the method, we consider a ‘kite’ object with homogeneous Dirichlet boundary conditions and make it appear as a ‘flower’ object with identical boundary conditions. Here, the field *u*_*i*_ is generated by a point source at *x* = (0.25, 0.5) and *t* = 0. For the heat equation, we took *k* = 0.2 and the domain is $\Omega =B(x_{0},r)$ with *x*_0_ = (0.5, 0.5) and *r* = 0.25. We computed the fields on the unit square [0, 1]^2^ with a 200 × 200 uniform grid. The field *u*_*c*_ is found by approximating the integral (2.5) using the midpoint rule in time with 180 equal length subintervals of [0, 0.05] and the trapezoidal rule on ∂Ω with 128 uniformly spaced points. A more detailed explanation, including how the scattered fields are calculated, is in §5. Figure 12*a*,*b* shows the scattered fields from two different objects and figure 12*c* shows the mimicked scattered field. Because we are approximating the fields numerically, the field *u*_*i*_ + *u*_*c*_ is very close to zero in Ω, but not exactly zero. The errors (which are not reported here) are larger near the boundary and decay as we move outwards, as we observed in §2.

**Remark 4.1.**

Although we have only shown mimicking of passive objects, the same techniques outlined in remark 3.1 could be used to suppress the field generated by an active object, which is part of what needs to be done to mimic an active object.

## 5. A simple numerical approach to potential theory for the heat equation

The boundary representation formulae (2.5) and (2.13) can be expressed very efficiently in terms of the single and double layer potentials for the heat equation defined for *t* > 0 by

$$K_{0}(\psi )(x,t)=\int_{0}^{t}\;\text{d}s\int_{\partial \Omega }\;\text{d}S(y)[\psi (y,s)K(x-y,t-s)],\;x\notin \partial \Omega$$

and

$$K_{1}(\varphi )(x,t)=\int_{0}^{t}\;\text{d}s\int_{\partial \Omega }\;\text{d}S(y)[\varphi (y,s)\frac{\partial K}{\partial n}(x-y,t-s)],\;x\notin \partial \Omega ,$$

as well as the corresponding boundary layer operators given for *t* > 0 by

$$V(\psi )(x,t)=\int_{0}^{t}\;\text{d}s\int_{\partial \Omega }\;\text{d}S(y)[\psi (y,s)K(x-y,t-s)],\;x\in \partial \Omega$$

and

$$K(\varphi )(x,t)=\int_{0}^{t}\;\text{d}s\int_{\partial \Omega }\;\text{d}S(y)[\varphi (y,s)\frac{\partial K}{\partial n}(x-y,t-s)]\;x\in \partial \Omega ,$$

where φ(*x*, *t*) and *ψ*(*x*, *t*) are time-dependent densities defined on ∂Ω and *n*(*y*) is the unit outward pointing normal to the boundary at y∈∂Ω. A full derivation of these and other potential theory operators for the heat equation and their properties can be found in [39,47,56]. For a review of classic potential theory, see e.g. [47,52,57,58].

With the potential theory notation, the interior boundary representation formula (2.5), with zero initial condition, becomes

5.1 $$u_{\Omega }(x,t)=K_{0}\left(\frac{\partial u}{\partial n}\right)(x,t)-K_{1}(u)(x,t).$$

Whereas the exterior boundary representation formula (2.13) becomes

5.2 $$v_{\Omega }(x,t)=K_{1}(v)(x,t)-K_{0}\left(\frac{\partial v}{\partial n}\right)(x,t).$$

Galerkin methods are commonly used to approximate the spatial integrals in equations (5.2), (5.1), with a number of different approaches to deal with the integration in time. For instance, time marching [38], time–space Galerkin methods [41,51], convolution quadrature [50] and collocation [59]. For simplicity, we opted for an approach based on the trapezoidal rule for the integration on ∂Ω and the midpoint rule for the time convolution. To be more precise, there are two convolutions that need to be evaluated in order to calculate the boundary representations in (5.1) and (5.2): a convolution in space and a convolution in time. For the spatial integration, the trapezoidal rule is used with uniformly placed points on a parametric representation of ∂Ω. Since this amounts to integrating a periodic function, we can expect that the convergence rate of the trapezoidal rule depends explicitly on the rate of decay of the Fourier coefficients of the function to be integrated [60]. Due to the smoothness of the heat kernel, exponential convergence is expected for smooth data and smooth ∂Ω. For the integration in time, the convolutions are of the form

$$\int_{0}^{t}\;\text{d}s\;g(s)f(t-s).$$

These convolutions are approximated with the midpoint rule as follows

5.3 $$\int_{0}^{j\delta t}\;\text{d}s\;g(s)f(j\delta t-s)\approx \delta t\sum_{k=1}^{j}g\left(\left(k-\frac{1}{2}\right)\delta t\right)f\left((j-k)\delta t+\frac{1}{2}\delta t\right),$$

where *δt* is the time step. The midpoint rule avoids evaluating *f* at *t* = 0. This is handy in our case because of the singularity of the heat kernel at (*x*, *t*) = (0, 0), which only occurs for boundary layer operators. After this space–time discretization, the resulting approximations are by construction an evaluation on the space–time grid of a finite distribution of monopoles and dipoles located on ∂Ω. These distributions are smooth solutions of the homogeneous heat equation on any open set of $(R^{d}-\partial \Omega )\times R$. Moreover, lemma 2.8 (see equation (2.27)) ensures that they are bounded for *t* > 0 and x∉B(0,r) for *r* large enough. Thus, as they satisfy equation (2.30), we can apply the maximum principle over any finite time window [*t*_1_, *t*_2_] (with 0 ≤ *t*_1_ < *t*_2_) on any closed set of $R^{d}$, with a bounded Lipschitz boundary, where the set does not intersect ∂Ω. We leave an accuracy study of the numerical approximation we use to future work. In particular, there are more accurate ways of dealing with the approximation for *s* ∈ [0, *δt*] than the midpoint rule we use, see e.g. [38,41,59,61].

### (a) Scattered field computation

In the case of inclusions, finding the scattered field requires the use of the boundary layer operators. For simplicity, we only consider a homogeneous Dirichlet inclusion, *R*, i.e. where the temperature on ∂*R* is held constant at 0. Neumann inclusions, and inclusions with varying thermal diffusivity, require the introduction of other boundary integral operators (adjoint double layer and hypersingular [39]), but a similar numerical approach can be applied to this case.

The idea is to look for a scattered field *u*_*s*_ of the form (5.2) outside of *R*. Since the temperature at ∂*R* is constant and equal to 0, the scattered field satisfies *u*_*s*_|_∂*R*_ = −*u*_*i*_|_∂*R*_. Hence, we know the Dirichlet data on ∂*R* in the representation formula (5.2), but not the Neumann data. We treat this as an unknown boundary density *ψ* in

5.4 $$u_{s}(x,t)=K_{1}(-u_{i}{|}_{\partial R})(x,t)-K_{0}(\psi )(x,t).$$

The latter formula is only valid for $x\notin \bar{R}$. To obtain an integral equation on ∂*R*, we take the limit of (5.4) as *x* approaches ∂*R*. The limit could be different if we approach ∂*R* from the inside or from the outside. The limits are given by the so-called jump relations. For *x* ∈ ∂*R*, the jump relation needed for the single layer potential is

5.5 $$\lim_{z\to x}K_{0}\psi (z,t)=V\psi (x,t),$$

which holds for *z* tending towards ∂*R* from both the interior and exterior of *R*. The jump relations needed for the double layer potential are

5.6 $$\lim_{z\to x^{+}}K_{1}\varphi (z,t)=\frac{1}{2}\varphi (x,t)+K\varphi (x,t)\;\text{and}\;\lim_{z\to x^{-}}K_{1}\varphi (z,t)=\frac{-1}{2}\varphi (x,t)+K\varphi (x,t),$$

where *z* → *x*^+^ denotes approaching ∂*R* from the exterior of *R* and *z* → *x*^−^ from the interior. Using these jump relations and (5.4), we have

5.7 $$\begin{array}{rl} & \lim_{z\to x^{+}}u_{s}(z,t)=\lim_{z\to x^{+}}K_{1}(-u_{i}(z,t))-\lim_{z\to x^{+}}K_{0}\psi (z,t)\\ & \Rightarrow \;-u_{i}{|}_{\partial R}=\frac{-1}{2}u_{i}{|}_{\partial R}+K(-u_{i}{|}_{\partial R})-V\psi . \end{array}$$

Rearranging terms yields a boundary integral equation for *ψ*

5.8 $$V\psi =\frac{u_{i}{|}_{\partial R}}{2}+K(-u_{i}{|}_{\partial R}).$$

We discretize (5.8) as a linear system where the unknown is *ψ* evaluated on a uniform grid of ∂*R* and of [0, *T*]. The boundary integral operators V and K in (5.8) are discretized using the trapezoidal rule in space and the midpoint rule in time. For instance, V is approximated by a *MN* × *MN* matrix, which is lower triangular by blocks, with each block of size *N* × *N*. Here, *M* is the number of time steps and ∂*R* is approximated by a polygon with *N* sides:

5.9 $$V\approx \left[\begin{array}{cccc} V_{\frac{1}{2}} & & & \\ V_{\frac{3}{2}} & V_{\frac{1}{2}} & & \\ \vdots & & \ddots & \\ V_{M-\frac{1}{2}} & V_{M-\frac{3}{2}} & \cdots & V_{\frac{1}{2}} \end{array}\right].$$

The *V*_*i*_ are *N* × *N* matrices with entries (*V*_*i*_)_*jk*_ = ℓ_*k*_ *K*(*x*_*j*_ − *x*_*k*_, *iδt*), where *x*_*k*_ is the centre of the *k*-th segment of length ℓ_*k*_. Clearly, the matrix in (5.9) is guaranteed to be invertible if $V_{\frac{1}{2}}$ is invertible. Though we have not studied the invertibility of *V*_1/2_, we observe that it may become singular for a given spatial discretization if the temporal discretization is not fine enough.

## 6. Summary and perspectives

We proposed a strategy for active cloaking for the time-dependent parabolic heat (or mass, or diffusive light) equation. Similar to previous work for active cloaking for e.g. the Helmholtz or Laplace equation (e.g. modelling time-harmonic waves and thermostatic problems), our results rely on active sources coming from Green identities to reproduce solutions inside or outside a bounded domain. The idea is to use a source distribution on a closed surface to reproduce certain solutions to the heat equation inside the surface and the zero solution outside or vice versa. We give a growth condition which is sufficient to guarantee that a solution to the heat equation can be reproduced outside of a closed surface. We apply these theoretical results in four ways: interior cloaking of a source, interior cloaking of an object, source mimicking, and object mimicking. For the cloaking problems, the idea is to find an active surface that surrounds the object or source to make the object or source undetectable by thermal measurements outside the surface. In the mimicking problems, instead of making the object undetectable, we make the source or object appear as a different source or object from the perspective of thermal measurements outside the cloak. Our solution to these problems inherits the limitations of the reproduction method, namely that the fields must be known for all time on the active surface surrounding the object or source we want to cloak or mimic. However, the maximum principle guarantees some stability of our approach, as it is based on boundary representation formulae. This principle is a fundamental property shared by a large class of time-dependent phenomena described by parabolic equations, see [44], and does not apply to hyperbolic equations such as the wave equation. We illustrate our method with simple potential theory-based simulations that are consistent with the heat equation and thus allow us to interpret the numerical errors using the maximum principle. Our study was limited to the case where the initial condition is zero or harmonic. It may be possible to use (2.8) to cancel out the initial condition with a boundary integral, but (a) it is not clear whether this mathematical construct has a physical interpretation and (b) this representation formula is only valid inside a bounded domain. These are questions we plan on exploring. Although we focused on the isotropic heat equation, it may be possible to carry out a similar cloaking strategy for anisotropic media and when an advection term is added to the heat equation. Our approach could also be tied to the active cloaking strategies for the Helmholtz equation in [13,14] by going to Fourier or Laplace domain in time, so that the active sources do not completely surround the object to achieve partial cloaking. For the Fourier domain, the physical interpretation is to study time-harmonic sources. Another open question is whether the growth condition we provided is optimal and how it is related to the uniqueness question for the exterior problem associated with different boundary condition types for the heat equation.

We note that this work could also be adapted to cloaking [62,63] and mimicking [64] of quantum matter waves. We believe this approach could be further generalized to the Fokker–Planck equation arising in statistical mechanics, which could be applied to gravitational systems, for which a passive cloaking theory has been proposed [65]. Finally, as a scattering cancellation-based cloaking approach has been proposed for Maxwell–Cataneo heat waves [66], we believe that our method for active cloaking could also be applied to such pseudo-waves.

## Data accessibility

We provide the following electronic supplementary material. (i) A PDF document with simulations for the one-dimensional reproduction problem and for the case with non-zero initial conditions. (ii) A movie (atc_movie.mp4) animating figure 10 (electronic supplementary material). (iii) The Matlab code to reproduce the figures 3, 5, 7–12 is provided in repository [42]. For example, figure 3 can be reproduced by running the Matlab script fig3.m. The scripts for figures 3 and 5 generate additional plots that are mentioned in the respective figure captions. The spatial distribution for the monopole/dipole added noise (§2.c) can be generated with the scripts fig9supint.m and fig9supext.m. Code for the figures in (i) is also included in [42], please see README for more details.
